# The Sailors’ Home and moral regulation of white European seamen in nineteenth-century India

**DOI:** 10.1080/14780038.2021.1901354

**Published:** 2021-04-01

**Authors:** Manikarnika Dutta

**Affiliations:** Oxford Centre for the History of Science, Medicine and Technology, University of Oxford, Oxford, UK

**Keywords:** Christian missions, colonial India, white seamen, philanthropy, Sailors’ Home

## Abstract

This article examines the efforts of British Christian missionaries in regulating the daily lives of European seamen in colonial Indian port cities. Missionaries aimed to reform seamen, who typically lived a life a debauchery and degeneration, by moral regulation and promotion of healthy living and hygienic practices in their everyday lives. Through a study of the Sailors’ Home run by Baptist missionaries and several other charitable institutions in Calcutta, this article provides new insights into maritime history, missionary history and colonial politics. Missionaries conceived seamen as not only the victims of government apathy and a malevolent labour market, but also tropical climate and an unforgiving urban space. This perception, rooted in the Victorian notions of purity and the link between a sanitary body and a moral mind, helped them justify regulating seamen’s bodies across the colonial world and beyond. The article shows how their actions produced white imperial bodies in a colonial context.

## Introduction

On 24 February 1838, the state of British merchant seamen was discussed in a meeting held at the Town Hall, Bombay. The speakers, mostly naval officers, largely concurred that discipline in the Mercantile Marine was in decline, making seamen demoralised, degraded, and uncompliant. One of the speakers, Sir Charles Malcolm, superintendent of the Indian Navy, said that in their current state, seamen greatly differed from the ‘former character of the British sailor for orderly conduct and obedience to discipline.’^1^ Echoing the sentiment in 1866, the Consul General of the United States of America for British India, Nathaniel P. Jacob, wrote to Major G.B. Malleson of the Bengal staff corps, ‘the active, quiet, respectful seamen of a quarter of a century since is now rarely met with.’^2^ Merchant seamen’s behaviour was a matter of concern throughout the history of British colonialism in India. White European recalcitrance imploded white racial superiority, which was one of the bedrocks of British imperialism. ^3^ British colonial authorities were concerned about instilling discipline among soldiers and seamen, but seamen usually escaped their attention. This is probably due to the diminishing of seamen's military importance for imperialism with the reduction in naval warfare and exploration. This article demonstrates that Christian missionaries in India took a leading role in reforming these seamen’s body and mind as part of their philanthropic activities, with the agenda of making them both good Christians and efficient imperial operatives. Baptists pioneered this field of missionary activity in the 1820s, and Methodists and Benedictines started their own philanthropic establishments for seamen in the last quarter of the nineteenth century. Much of these activities took place in Calcutta, the capital of British India and the largest sailortown in South Asia in the period.^4^ Through a study of the Sailor’s Home and ancillary missionary work among white European seamen in Calcutta between the 1820s and the 1890s, this article offers three original insights that deepen the existing understanding of maritime labour and religious philanthropy in colonial India.

First, it recentres colonial maritime history’s thematic emphasis on Asiatic seamen (colloquially known as Lascars), mobility, labour, recruitment and port administration to issues of white European seamen and moral regulation.^5^ It builds on the works of historians Harald Fisher-Tiné and Sarmistha De on European seamen in Calcutta as a group of highly visible insubordinate white subalterns, whose actions subverted the British colonial state’s idioms of racial and ideological superiority.^6^ Fisher-Tiné and De have explored the colonial government’s anxiety about the high rate of unemployment and mortality among seamen in Calcutta in the 1850s and 1860s due to the disbanding of the armed forces after the Indian Mutiny of 1857, large scale desertion and reduced recruitment. Most of these seamen became vagrants and some resorted to crime as a means of livelihood. Going beyond the colonial state’s relief measures for these seamen, this article examines the activities of Baptist missionaries as a non-state actor over a longer period, expanding our understanding of how philanthropy served imperial interests and class divisions in maritime spaces. The emphasis on non-state actors also serves as a counterpoint to existing historical research that has focussed on the colonial state’s moral regulation and the Salvation Army’s work among Europeans in India, most notably the disciplining of soldiers in the army cantonments and ‘poor whites’ in general.^7^

Second, the article expands the historiography of ‘maritime missiology’ by adding India as a new context of research. Pioneered by Roald Kverndal in the 1980s, studies of maritime missions have examined the origin and spread of seamen’s welfare organisations mainly in Western Europe and North America. They have considered various aspects of seamen’s and fishermen’s missions and welfare provision, including detailed studies of Sailors’ Homes, individual missionaries and organisations, the nature of evangelicalism, and humanitarian reform in Britain.^8^ In these works, the relationship between religion and identity in seamen’s lives has foregrounded the seaman as an object of politics and charity. With its focus on the theme of reclamation of European seamen’s morality as a means of transforming them into ideal Christians and productive workers, this literature largely overlooks how these missions served a regulatory function by treating seamen as sanitary subjects. This article sheds light on the challenges of regulating seamen in a British colony amidst threats of racial debasement and tropical diseases, and explores what the ways of addressing the situation tell us about the missionary enterprise in the nineteenth century.

Third, the spaces of missionary-seamen interaction offer a new perspective to understand colonial missions. Missionary activities in colonial India are usually analysed as examples of primarily white people intruding into non-Christian life, denigrating indigenous customs, imposing foreign cultural conventions, gathering local knowledge mostly to denounce it, and building schools and other charitable institutions with the motive of ultimately converting locals, often through violence.^9^ However, the interaction with marginal Europeans suggests an alternative history of missionary work in the colonies that is rarely referred to in the historiography. It demonstrates how Christian missionaries envisaged European seamen as victims of the apathetic East India Company and the colonial government, a malevolent labour market, tropical climate, and unforgiving Indian urban spaces that were full of disease and crime. It argues that seamen’s welfare through regulation of their bodies and habits illustrates the use of religion in resolving moral and medical anxieties around white people in the colonies. This study of Christian missionaries enriches the works on problematic imperial whiteness that have shown how British government officials and intellectuals created imperial imaginaries of race and belonging.^10^

Based on a study of the Sailors’ Home in Calcutta, the second oldest such institution in the world after London (1828) and built in the same year as New York (1837), the article argues that moral preaching and promotion of healthy living and hygienic practices were not aimed at simply reforming seamen, but also governing the spaces of vagrancy, disease and epidemics that the British imperial government struggled to do by itself. It unpacks evidence from missionary books, tracts, annual reports, and periodicals from India, Britain and the United States, newspaper reports from India and Britain, and records of the Government of India. It circumvents of the problem of the self-serving nature of missionary documents by using memoirs and reports that provided insider perspectives into the inadequacy of the missionary enterprise. Reports of the Government of India from the British Library’s India Office collections likewise offer detailed descriptions of seamen’s problems and the extent of missionary and government interventions. This eclectic assemblage of sources enables the article to examine how missionaries exerted a specific iteration of the non-state dimension of regulation, which worked separately from other technologies of power such as penal and disciplinary power.

## The need for moral regulation and a Sailors’ Home in Calcutta

Seamen’s missions grew out of an ecumenical obligation of spreading the gospel. Missionaries understood that use of rhetorical devices alone will not help them bend seamen to the will of God, so they started offering tangible benefits. Therefore, in addition to lectures and tract distribution, Baptist clergy built Sailors’ Homes in major port cities across the world. These establishments recreated an amiable social structure usually absent from seamen’s everyday life and sought to transform the lives of white seamen (and rarely of non-white seamen).^11^ Clergy of various other denominations too preached the gospel among seamen and promoted temperance and appropriate male behaviour as part of their social work. Their work represented a paternalistic attitude that spiritual reawakening was more powerful than the rule of law.^12^ The conviction that they knew what was best for the allegedly uninformed and errant Christian seamen was grounded in the hierarchal relationship between the lowly working class and the superior men of God. Placing themselves above political institutions, whom they alleged were not supporting seamen and the downtrodden in general, the churchmen sought to establish themselves as the highest moral authority of the imperial nation. They treated the Sailors’ Home as a regulatory institution. This attitude was encapsulated in religious writings and speeches. For example, in their eighth annual meeting held in 1841, the British and Foreign Sailors’ Society in London stated the aim to:

… forestall and destroy those influences which operate to the ruin of our home population … seeking out the suffering sailor, at home and abroad, to place him in the hospital, to watch over him there, soothing his sorrows, administering medicine and consolation, and above all, pointing him to the great physician … through the moral purity of our seamen, as an important instrumentality, that monster, whose breath has poisoned the institutions of so many nations … shall relax his grasp … ^13^

These visions were not held exclusively by members of the clergy. Naval officers, merchants and politicians were also part of societies for seamen’s welfare, though the extent of their involvement and authority over operations is hard to determine. Missionary documents rarely praised civilians. In the early to mid-nineteenth-century, moral reform was a significant part of sanitary reform in the British Empire. The theories of disease prevalent in this period espoused the need for a holistic improvement in physical and moral health. These considered disease to be the result of both ‘discrete external stimulus’ and the body’s fight to restore the normalcy endangered by personal habits.^14^ As a person’s moral character was increasingly aligned to health, Christian clergy had a justification to evangelise their ideas of moral purity. In stories told during sermons, the Bible often became a medicine to help seamen recuperate from illness. One such tale, recounted by a clergyman in Calcutta, related to a young sailor whose mother gave him a Bible on the eve of his voyage. The book lay forgotten at the bottom of his chest till he fell ill and started reading to overcome his depression.^15^ The gospels thus became a panacea for mental wellbeing. In addition to sermons, paternalistic ideas were put into action through Sailors’ Homes. These institutions, as is evident in the use of the term ‘home’, provided seamen a respectable surrogate family of fellow seamen to deter them from visiting pubs and brothels. They replicated the YMCA’s principles of ‘domesticity, discipline, and sobriety’ with greater religious overtures.^16^ The Sailors’ Home sought to transform reprobate seamen into hardworking respectable citizens who would avoid both mixed-race and rough homosocial pastimes.

The initiative to build a Sailors’ Home in Calcutta was taken by Rev. William Ward, a highly influential Baptist missionary who managed the printing of Christian scriptures in Indian languages and co-established a college in Serampore. He had attended the anniversary of the Seamen’s Friend Society at the City of London Tavern in 1821 and addressed a gathering of Lascars in Bengali at the Mariners’ Church.^17^ On his return, he made a futile attempt to start Sabbath services for European seamen at the Bow Bazar Chapel in the autumn of 1821. Undeterred, he established the Calcutta Bethel Society at a public meeting held at the Union Chapel on 4 June 1822. After initially struggling to convince ship captains to send their crew to the chapel for prayer, the Society purchased and repurposed a boat as a floating chapel for 150 people. Named Calcutta Ark in direct tribute to the London vessel, it was inaugurated on 27 July 1822 with a sermon by William Carey, co-founder of the Baptist Missionary Society (1792).^18^ The boat was anchored opposite the Metcalfe Testimonial on Strand Road.

The Society next started a Bethel Lodging House to keep European seamen away from the taverns filled with risqué banter and pernicious liquor. It was soon closed due to the lack of a ‘faithful’ superintendent.^19^ Early reports of these institutions indicated a general lack of public interest. The Calcutta Seamen’s Friend Society was established in 1827, the same year as the Boston Seamen’s Friend Society and a year before the American Seamen’s Friend Society in New York. Its objective was to preach the gospel, distribute religious tracts and books to seamen, and attend to their spiritual needs. The Society for the Propagation of the Gospel in Foreign Parts, the overseas arm of the Church of England, sponsored the Anglican mariners’ church that was opened on 16 May 1830.^20^ Missionaries soon realised the need to open a subsidiary institution that would offer more tangible benefit and, hence, attract seamen to Christianity. They were greatly troubled by seamen’s failure to rise above ‘savage’ Indians on account of their violence and immorality, which also undermined the ideal of British/white national and racial supremacy.^21^

These measures led to the establishment of Sailors’ Homes in Calcutta, Bombay and Madras. The Secretary to the Calcutta Seamen’s Friend Society, Rev. Thomas Boaz, convened a meeting on 1 February 1837 at the Union Chapel House in Central Calcutta to discuss the feasibility of establishing a Sailors’ Home.^22^ Three resolutions were unanimously adopted at the meeting: there was need for an institution to protect seamen from social evils; the institution must be managed judiciously and efficiently; and a committee would be appointed to execute the motion. Rev. Boaz chaired a subcommittee that drafted a prospectus for a Sailors’ Home. It was circulated among European merchants and civilians in the city in the hope of raising fund and government aid.^23^ The text said that seamen, easy preys of unscrupulous men, were in greater trouble in the colonies in the absence of a nurturing society to protect them. Indulgence in ‘vices’ destroyed their health and character. If left unchecked, their misery would soon escalate, affect maritime enterprises, and shatter ‘domestic happiness at home’ i.e. the British metropole.^24^ An apparent lack of interest of British imperial naval authorities in maritime health, sanitation, security, and employment reforms in particular both forced and provided justification for missionaries to act.^25^

Once seamen reached an Indian port and ventured on shore, they were approached by people recommending the best places to eat, drink, stay, and liaise with local prostitutes. These people were known by the name crimp, a ubiquitous feature of any port in the world, who were defined as recruiters in the maritime labour market.^26^ Crimps guided seamen to dens of drinking and entertainment. A majority of crimps were Indian boatmen, colloquially known as *dinghywallahs*, who were hired by the commanders of merchant ships to run errands and ferry seamen to the shore.^27^ Their ability to speak broken English, combining words learnt from seamen without any real order, containing mainly swear words, endeared them to the ship’s crew. Recounting his experience in the 1820s, Baptist missionary John Statham wrote that boatmen were ‘one of the many harpies which pounce upon [the sailor].’^28^ They offered to be seamen’s guide and interpreter when the latter went ashore, dressed in a ‘white jacket and trowsers, with new straw hat, tied round with a blue ribbon.’^29^ They ushered seamen to waiting palanquins, taking a commission from the bearers, and took them to grog-shops, usually in the Old China Bazaar. The next morning, the seamen would find themselves penniless and miserable, possibly lying on a sidewalk. They would persuade or threaten a boatman to take them to their ships and spend the next week sulking about the whole incident. In other reports, Indian crimps were said to have been ‘victimising,’ ‘inveigling,’ and ‘seducing’ seamen who intended to return to their ships after spending days on shore. They persuaded seamen to desert ships with the lure of more wage or a new job in country or coastal trade. While waiting for the next job, seamen indulged in ‘vices’ and were ‘totally destroyed by the dissipations’ – becoming very different from the person who had begun the journey in full ‘bloom and vigour of health.’^30^ As one ship captain was quoted saying in the *Sailors’ Magazine*, ‘thus is the most noble and most generous of Britain’s sons duped, before he sets his foot ashore … ’^31^

Indian crimps were not too different from the British crimps in the seamen’s quarters in London and Liverpool.^32^ The crimps in Britain took seamen to boarding houses, tap-rooms of public houses, long rooms of gin-palaces, and brothels. An anonymous writer to the *Sailors’ Magazine* blamed seamen for falling into well-known traps, and ship-owners and captains for insensitively driving sailors towards destitution.^33^ Crimps persuaded seamen to stay at specific lodgings, buy articles from shops of their choice, and drink at pubs that gave them commissions. The propensity to relax in the port city after too much hard work and lack of recreation on board vessels led to drinking binges. An inebriated sailor could be easily persuaded to turn to crime and a ‘libertine’ life. The writer refers to ‘foes’ who drugged the drinks of seamen who risked becoming addicted, ‘enslaved’ to the ‘virile’ poison. By the time the sailor had recovered from his drug-induced reverie, these crimps would have disappeared with all his belongings. Unable to afford meals and accommodation, the sailor depended on the same or another crimp for loan. The *Friend of India* wrote that the condition of seamen in Calcutta was no different from those in Liverpool, Glasgow, Hull or Plymouth, who indulged in the same recklessness and vice.^34^ However, compared to crimps in England, who apparently tempted sailors to ‘brilliant dancing rooms, rich in gas light, and society rendered fascinating by pink ribbons, red paint, and such music as is permitted to a harp and two fiddles’, the Bengali crimp did disservice to seamen by offering him a life in the ‘squalor’ and ‘repulsiveness’ of the dens of Calcutta.^35^ The materiality of deception was also an tool for the newspaper to comment on the relative opulence of Britain in comparison with India.

Crimps had a second function as employment agents. They carried around ‘able seamen wanted’ placards. Their clients were, on the one hand, seamen who had lost or spent all their belongings during nights of revelry on shore leave, and deserters, and, on the other hand, ship captains who needed fresh crew to replace dead or deserted crew members. Crimps lent money to destitute seamen for exorbitantly priced accommodation and clothes. They took commission from unscrupulous ship-owners for recommending these seamen as cheap labour at short notice. ‘Drugged,’ ‘stupefied,’ distressed seamen could not bargain for a proper wage.^36^ Moreover, they had to repay the crimp’s loan with a high interest. In Calcutta and Bombay, unemployed seamen in large numbers desperately filled in for deceased or debilitated seamen for a meagre compensation, much to the benefit of crimps. In missionary accounts, these seamen were deceived by these ‘cunning and villainy’ ‘native inhabitants’ whom they thought were ‘simple and ignorant.’^37^ It should be noted that unlike what Christian missionaries reported, there were many English and American crimps in India, most of whom were tavern owners and more adept at communicating with European seamen due to the lack of a racial and language barrier. One of them, Bennet Braham, while testifying for a ship captain at the Supreme Court in 1824, revealed that he owned a tavern for which he paid Rs 150 a month as tax, and he had been a crimp for 6 years. He boasted that he shipped more seamen than all other tavern keepers put together.^38^ His unabashed statement in a court shows that he considered himself to be a labour supplier rather than a criminal, and the moral implications of crimping was mainly a missionary complaint.

Many of the European seamen who deserted ships turned to a life in organised or petty crime. In 1788, The Governor-General of India received several complaints of disorderly behaviour by Portuguese and other European origin seamen in Calcutta. On 23 April 1789, Marine paymaster J. Price wrote to E. Hay, Secretary to the Government at Fort William, ‘money paid into [seamen’s] own hands serve[d] only to make them more troublesome, get drunk and lay about the streets, and ultimately die in the hospital … ’^39^ Hay asked the captains and masters of ships anchored in Calcutta to ensure their crew would not come on shore before seven in the morning and leave by five in the afternoon.^40^ He specifically targeted non-white seamen, ordering arrest of any ‘Portuguese, Coffrees, Manilla or Macoa men, or Malays’ sailor found in the city outside these hours. Yet, the reports of seamen’s crimes almost invariably involved European seamen. Two Europeans who assaulted a gentleman during his evening walk on a Sunday in 1790 appeared to be sailors from the way they dressed.^41^ In 1794, a gang of nine or ten European seamen attacked a band of sepoys and looted the treasure they were escorting. In an attempt to protect national prestige, a correspondent emphatically mentioned that none of the apprehended seamen were ‘English.’^42^ However, in 1857, after a fight among some Highlanders and sailors in a liquor shop opposite a police station, the reporter wrote with dismay that a group of English men were causing such commotion and tarnishing Britain’s reputation.^43^ Some European seamen formed multinational gangs that operated in the area between the ‘opium dens and whore houses’ of Chitpur in the native quarters and the taverns in Lal Bazar that were between the white and native settlements in the city.^44^ Many criminals arrested from this area were identified as British soldiers posted in the Fort William, and English, French, Italian, Spanish and Portuguese seamen deserters.^45^ The missionaries, taking responsibility for the moral uplift of white imperial seamen, built the Sailors’ Home as an institution of reform.

## European seamen as moral subjects

According to the prospectus, the Sailors’ Home would provide European seamen with corporeal and moral needs – ‘comfortable lodging, plain food, innocent recreation, and religious guidance’. It would have two branches: the boarding – a respectable and economical boarding house for the paying captains and sailors; and the destitute – to shelter ‘shipwrecked, convalescent, or otherwise distressed’ seamen till they could be shipped off to their homes. The Calcutta Seamen’s Friend Society would maintain a register of crew requirement of anchored ships and advise boarders about job opportunity. They would keep seamen’s money for safekeeping during their stay to prevent them from spending on ‘trivial pursuits.’ A library would be established, and boarders provided with pen and paper for writing letters. The Committee were to decide the means of ‘temperate enjoyment’ suitable for seamen. Other recommendations included rewarding seamen for good behaviour at the Sailors’ Home and onboard ships, lending libraries for ships visiting Calcutta, recruiting a visiting and a permanently resident superintendent, and appointing missionaries to visit ships for publicising the Society’s actions. The visiting superintendent would preside over daily religious service. The Society sought to enforce temperance and ‘ship-discipline’ in regard to food, drink, the use of liquor, daily routine and conduct. Finally, the committee to manage the Sailor’s Home would be drawn from the pool of subscribers, and all captains and officers staying there could be ex-officio members of committee on paying the subscription fee.^46^ Missionary records do not mention the size, shape and decoration of the rooms. Indeed, missionaries intended to articulate ideas of domestic life to create docile bodies, but sources are silent on how these domestic ideals were exercised through material objects and the architecture of the Sailors’ Home.

The Committee estimated a monthly expense of 600 rupees for sheltering 40 men. The expenses for the building, the furniture and running costs would be covered by the rent from the boarding section, and donations from magistrates, merchants, shipping agents and captains connected to the port of Calcutta. Rev. Boaz presented the idea of the Sailors’ Home in a public meeting at Town Hall on 18 March 1837. In the meeting, one Mr Strettell delivered a speech about the threats of licenced punch-houses to unsuspecting seamen. The meeting was attended by many affluent merchants who promised to donate about Rs 4000 to the cause. The committee also discussed the feasibility of improving the condition of Lascars.^47^ Mention of non-white seamen in missionary reports from the period of this study is otherwise rare. Missionaries usually defined the problems of European seamen in racialised terms, describing of Indian crimps and local food and drink to be particularly dangerous.^48^

The committee opened a small house in Jan Bazar in June 1837 as a short-term residence for around 30 destitute seamen. That year, a total of 981 British and American vessels with 14,417 seamen, besides Lascars, arrived in the city. Among the seamen given shelter on the first month were the crew of the ship *Rebecca* that was wrecked on the Coromandel Coast. Most of the boarders were helped to find ships quickly.^49^ The Sailors’ Home was built later in the year at the site of the Harmonic Tavern in Lalbazar, a popular destination for seamen in the late eighteenth century. The programme for the inauguration ceremony noted that the establishment was intended to ‘suppress crimping and all the evils arising from it to which owners, commanders, officers and crews are subject in the port of Calcutta.’^50^ British people coming to Calcutta usually had letters of introduction that enabled them to stay at certain places. Soldiers stayed in barracks, merchants lived with their correspondents, and ship captains went to their friends’ houses or rented accommodation. Sailors and junior officers in merchant ships headed for taverns where they spent a lot of money for recreation. The Sailors’ Home sheltered these seamen, with separate accommodation provided for quarter-deck officers and other officers.^51^ The house contained a library and reading room.^52^ The opening of the Calcutta Sailors’ Home set in motion a building spree that saw the establishment of similar institutions in Bombay, Madras and Penang in the same year.^53^ The Sailors’ Home was shifted to Captain Birch’s spacious house in Lal Bazar in 1842.^54^

*The Calcutta Christian Observer* wrote that the Calcutta Seamen’s Friend Society should strive harder to abolish the crimping system. It argued that a comparison between the numbers of sailors provided jobs by the Sailors’ Home and crimps, or sailors finding their way to the Sailors’ Home and to crimps, would illustrate the success of sailors’ welfare measures. It suggested the managers of the Sailors’ Home should seek police cooperation to dispose of crimps, ask shipping lines to give jobs only to seamen sent by them, and employ a number of agents for visiting ships on their arrival and shepherding sailors to the Sailors’ Home.^55^ The *First Half Yearly Report* of the Calcutta Sailors’ Home emphasised the need to counter ‘drunkenness, plunder, prostitution, violence and quarrels.’ To this end, the Sailors’ Home authorities aided the destitute seamen with subsistence and clothes, safeguarded deposits of wages, helped them get jobs in departing ships, encouraged sobriety and good behaviour, and preached the values of Christianity.

Apparently, the residents were full of praise for the services. Appended to the report were two letters of gratitude to the Secretary from the crews of the *Abberton* and *Sesostris* and one from a destitute sailor. This could be genuine notes of appreciation, but the self-congratulatory disposition of missionary publications and the tendency of humble subjects to write deferentially to people in power must be considered when treating such texts as evidence.^56^ In the first six months, the Sailors’ Home admitted 303 officers and seamen, of whom 264 were provided with berths. There were two deaths at the shelter and only 20 people remained as residents at the end of the year.^57^ A shipping agent tried to find work or passage for the resident seamen. Inmates were allowed to stay for a maximum 25 days. The Sailors’ Home authorities watched their health and hospitalised who were ill for over three days. Meals were the same price as in other cities. Meals for the entire day cost 12 annas, breakfast and supper 4 annas each and dinner 6 annas if bought separately. Officers needed to pay 8 annas for every meal. The bar supplied liquor from 6 to 7 am, from 12 to 2 pm, and 5 to 9 pm on weekdays, and in Sundays from 1 to 3 pm.^58^ The number of sailors accommodated by the Sailors’ Home in 1852 was 1419, out of whom 1381 took jobs on ships, 24 died, and 28 left the place (4 without employment, 6 without notice, 10 were expelled, and 8 shifted to new occupation) without leaving any track.^59^ The annual reports were usually congratulatory of the Sailors’ Home’s efficiency.

The necessity of regulating seamen’s health received an impetus after the Indian Mutiny of 1857 when the British philanthropist Florence Nightingale started writing petitions to the British government for improving the health of troops. The mortality records of soldiers convinced her that lack of sanitation was a severe threat. In a report entitled *Observations on the Evidence Contained in the Stational Reports Submitted to Her by the Royal Commission on the Sanitary State of the Army in India*, prepared in 1863, she advocated for improving sanitation in barracks, building hospitals in the cantonments, and providing a coffee room, a theatre, a gymnasium, and a reading room in the barracks to develop the morality of soldiers.^60^ In a series of letters to Douglas Galton, Assistant Under-Secretary to the War Office, in early 1864, she criticised the Admiralty and War Office for devising the wrong diet on board ships that led to scurvy among troops, requesting Galton to ask the chairman of the Barrack and Hospital Improvement Commission about the general principles of rationing at sea.^61^ In her correspondence with the Viceroy of India Sir John Lawrence, she expressed concern about poor quality food, water and drinks causing disease and death among seamen in Calcutta, where establishing more Sailors’ Homes seemed to be a way of saving them.^62^ The Government of Bengal showed more interest in seamen’s health in the 1860s than ever before. After the Floating Chapel and Seamen’s Library were destroyed in a cyclone on 5 October 1864, they sanctioned 9000 rupees to convert the steamer named *Lady Canning* into a new chapel.^63^ In the library, seamen were provided with periodicals, books, newspapers, writing materials such as papers as well as postage stamps to encourage them to write letters to home, to help them bond with their families and not to feel lonely during their sojourn.

The popularity of the Sailors’ Home as a refuge was certainly an established factor, which led to the building of several other institutions for housing more seamen. A second Sailors’ Home in Calcutta began to be planned in 1850. It was reported that more than £4,000 had been raised by 1851 for this purpose.^64^ The plan materialised when the Secretary of the Sailors’ Home successfully petitioned the Lieutenant-Governor of Bengal in 1864 for a new building in a better locality. He also expressed the need for a recreation ground, enclosed with a bamboo fence, resembling a cricket ground.^65^ The Bengal government allowed enclosure of a part of the vast field next to the Esplanade for seamen to play cricket.^66^ The new Sailors’ Home was built on the site of the Bankshall building on Strand Road in the city’s administrative district. The Viceroy laid its foundation stone on 13 April 1864.^67^ The building’s initial cost was estimated at 34,388 rupees but was finally completed at an expense of 173,000 rupees. Sir John Lawrence inaugurated the building on 9 January 1868. Designed by W.L. Granville and planned by W. Barnfather of the Bengal Public Works department, the two-storey building offered accommodation for 120 on the ground floor and 160 on the first floor, while the dining hall could hold 600 men.^68^ But what exactly happened inside the premises? Were they really able to solve the problem of destitution, convince seamen not to desert ships, or turn them away from drinking and visiting prostitutes?

## Regulation of seamen’s habits and everyday life

Missionary Rev. T. Smith thought all the seamen he met at the Calcutta Medical College Hospital were responsible for their ailments – seamen themselves and crimps were more dangerous than hurricanes and the capsizing of ships; and seamen needed the help of missionaries.^69^ Several reports from the early- to mid-nineteenth century extolled the growing Christian influence on seamen’s behaviour. Such publications had their own politics of religious sectarianism. A report in the *Sailors’ Magazine*, published by the American Seamen’s Friend Society, said that the Roman Catholic captain of *Ramsay*, which sailed from Glasgow to Bombay in 1840, and subsequently to Moulmein and Calcutta, inspired his crew to be sober and regular attendees of the floating chapel.^70^ In a letter dated 27 July 1844, the President of the American Seamen’s Friend Society, E. Richardson, congratulated the Calcutta chapter on their excellent work, and requested information about the work of missionaries in Asia, especially in regard to Sailors’ Homes.^71^ At the 17^th^ annual meeting of the Calcutta Seamen’s Friend Society in 1845, missionaries were thus reminded of their humanitarian goal:

The Christian Church, in its collective and especially in its missionary capacity, owes much to our seamen, both in the way of gratitude and responsibility; gratitude, for conveying to different parts of the world her heralds of salvation, responsibility, in looking well to this that they mar not by their guilty example the work of God in foreign lands.^72^

At the 22^nd^ annual meeting of the Calcutta Seamen’s Friend Society in 1851, the missionary Mr Chill reported to have distributed 431 Bibles and Testaments and 4607 tracts and books in various languages to 375 vessels all year. The number of seamen attending the Bethel was on the rise, so was the Society’s fund to establish a mariner’s church.^73^ Self-congratulation was a feature of missionary writings. In a letter to the *Sailors’ Magazine* in 1856, S.E. Bishop, Seamen's Chaplain in Lahaina, Hawaii, wrote about massive improvement in seamen's behaviour across the globe – in Melbourne, Calcutta, and Valparaiso.^74^ If one were to look into missionary records exclusively, European seamen would come across as a reformed collective of patriots. A ‘seaman’ alleged that missionaries were more welcoming than the general Christian population who were usually uncomfortable in the presence of uniformed seamen.^75^ Missionaries claimed to have helped improve seamen’s behaviour. Their propaganda machinery was ever so alert to point out their contribution, probably in the hope of attracting financial support and volunteers.

The Sailors’ Home regulated the lives and eating habits of their residents. The philosophy of missionary philanthropy for seamen in colonial India differed from the outlook towards the working class in Britain. Showing preference for ‘ministering to working-class souls rather than providing for working-class bodies,’ evangelists in Britain preached morality instead of providing assistance in the form of alms, health care, housing, food etc.^76^ They considered material aid to have a negative impact on the recipient’s will and willingness to work. This moral understanding of the nature of social work, implicit in British missionary thinking in the early nineteenth century, was only partially practised in India. The Sailors’ Homes offered complimentary accommodation and meals to many destitute European seamen to save them from imminent hunger, homelessness, and possible death on the streets. Therefore, the paternalism that the Christian clergy opposed in Britain became the preferred method of welfare elsewhere.

The Calcutta Sailors’ Home authorities took a sanitarian outlook on seamen’s health. They appointed a superintendent for cleanliness, who inspected the beddings seamen brought along.^77^ They ensured cleanliness of the premises as the beginning of their residents’ moral journey. The Committee also gave a certificate of character to seamen commending them for avoiding local grog shops and prostitutes. This letter of recommendation enabled them to find lodging in Sailors’ Homes in other port cities.^78^ One had to follow the expected norms of behaviour to be able to receive this character certificate. A necessary condition was observation of temperance. The Sailors’ Home authorities imposed a strict restriction on drinking. They did not allow residents to bring alcohol from outside or consume more than two glasses of alcohol in a day inside the premises. Barmen use their discretion to sell spirits to specific boarders. The Sailors’ Home served no alcohol if they were already inebriated, and the House Committee expelled residents who exceeded their quota of drinking.

Contrary to the Committee’s claim to have neutralised the threat of drunkenness, at least one official of the Sailors’ Home went on record saying the benevolent institution achieved precious little in this field. Rev. Thomas Atkins was appointed the Sailors’ Home’s Superintending Secretary within ten days of his arrival from Australia in 1838 and the Minister of the Bethel after a few more weeks. His memoirs are full of regret for having served in this capacity.^79^ He wrote that one of the benefactors of the Sailors’ Home was actively engaged in the sale of spirits, beer, and wine. About half of the members of the management committee were directly or indirectly involved in the liquor trade. They encouraged drinking in the Sailors’ Home, even if in a small quantity, to make the residents crave more liquor. They made little effort in stopping inmates from visiting public houses, becoming drunk, and committing crime. Atkins was often awakened by drunk seamen noisily scaling walls on their return from punch houses and brothels. Poor management had made the Sailors’ Home a curse rather than a blessing for seamen and ship owners.^80^

Atkins saw himself as a ‘licensed victualler’ whose main responsibility was to monitor the store of ‘beer, in barrels and bottles; wines – port, sherry, and claret, in dozens; spirits – brandy, gin, and rum, in large quantities.’^81^ He notified the general committee about the tendency of barkeepers to sell liquor above the limit and to steal alcohol from the store at night. As his proposal to close down the sale of liquor to avoid such problems was rejected by the committee, a humiliated Atkins resigned from his position after the 12-month term. At the next meeting of the committee, he raised the demand of total abstinence at the Sailors’ Home. His repeated entreaties led to an enquiry, conducted by ‘traffickers in alcoholic drinks’, which ultimately ruled in favour of selling alcohol.^82^ Two other clergymen associated with the Sailors’ Home resigned in protest. The disjuncture between policy and practice comes out from personal narratives such as Atkins’ reminiscences. The annual reports of the Sailors’ Home or proceedings of the annual meetings would not refer to such disagreement over the running of the institution, or any allegation of mismanagement against the governing committee.

A number of other observers were critical of the Sailors’ Home’s early failure to deliver its promise of moral purification. In the 1840s, young people leaving England full of hope and aspiration ‘buried’ their dreams in Lal Bazar and returned home, if they did, with ‘heart contaminated, his principles perverted.’^83^ A pamphlet published by the Calcutta Seamen’s Friend Association on the occasion of the Sailors’ Home’s tenth anniversary claimed that within 18 months the institution was able to orchestrate closure of all but one of the punch-houses in the vicinity, and the one still open did not usually have more than one occupant. Yet, as *Calcutta Review* reported, scores of punch-houses existed near the Sailors’ Home. They made so much profit that their owners did not hesitate to pay up to three rupees per day as licence duty. The minimum annual licence fee for a punch-house in Calcutta was 939 rupees or £93.18s, which the owner would pay without whining as rich attorneys in Britain were known to do.^84^ Evidently, the Sailors’ Home was not managed as efficiently as the missionaries claimed.

In its inaugural meeting at the Victoria Hotel in Calcutta in 1853, the Chairman of The Society of United Seamen for the Protection of Seamen of all Nations called the Sailors’ Home ‘the hotbed of misery.’^85^ Montague Massey, a civil servant, wrote that it was a ‘crying scandal’ in the 1860s, mainly due to its location in an area abounding with ‘native grogshops in which [shopkeepers] sold to the sailors most villainous, poisonous decoctions under various designations,’ and ‘boarding houses run by a thieving set of low-caste American crimps.’^86^ This is probably the only narrative that deviates from the racialised description of the ‘cunning’ Indian crimps. Norman Chevers, Principal of the Calcutta Medical College, a major temperance advocate, and one of the most important medical authors in the British Empire, too said that the Sailors’ Home was ‘surrounded with drinking shops of vilest description’ and situated in the ‘centre of about the worst atmosphere discoverable in this unsavoury city.’^87^ He suggested constructing a larger building in a more sanitary and ‘reputable’ part of the city. Neither did this happen, nor did the sale of alcohol in or around the Sailors’ Home ever stop.^88^ Seamen’s Chaplain A.L. Mitchell advocated for an institute for seamen to socialise, play bowling, chess, and draughts, and drink tea, coffee, ‘good’ soda-water, ginger beer and lemonade at proper rates.^89^ Reports of violence and drunkenness at the Sailors’ Home almost 50 years after they were conceived in India as a school for temperate lifestyle did not lend themselves well to supporting the missionaries’ claim of success. Moral regulation appeared to have fallen short of the expected outcome.

These narratives of negligence, corruption and inefficiency mirrored the complaints against Sailors’ Homes in Britain. David Beckingham’s research on the civil administration’s crusade against the sale of alcohol in Victorian Liverpool, ‘a city with a drink problem’, shows the Sailors’ Home at the very heart of the problem of intoxication and prostitution.^90^ In acceptance of city’s weekly political journal *The Porcupine’*s dismay in 1869 regarding the ‘licentiousness, harlotry, and drunkenness’ around the institution, the municipal administration launched an investigation and found the rackets of prostitution to be operating mainly from the drinking hubs. Although merely 5 to 8 percent of the people put under arrest for drunkenness were seamen, the constant exposure to liquor shops, brothels, and pawnbrokers was considered to have contributed to moral degradation among the seafaring community.^91^ As the Sailors’ Home was shifted to a new location in 1878, the authorities made sure they purchased the adjacent properties so that no grog shop could come up in close proximity. The threat of drunkenness made the municipal authorities consider ‘moral means’, uncertain if policing alone would be enough as a solution.^92^ The Portsmouth Sailors’ Home on Queen Street was also surrounded by a number of ‘beer-houses,’ which, reckoned one observer in 1854, were built with the purpose of leading seamen astray. In the report of the institution’s operation in the Religious Tract Society’s magazine, the ‘clean, intelligent-looking, respectable’ seamen in the Sailors’ Home were sharply contrasted with the ‘dirty drunken sailors staggering out of these taverns.’^93^ Thus, the sharp difference between missionary claims and observers’ reports was by no means confined to India but was rather a global pattern.

It is not possible to determine how effective the Sailors’ Homes in India were in eradicating drunkenness, but the policies indicate a clear intention of at least regulating drinking to smother what they considered an inherent vice in seamen. As potential clients of the so-called disreputable pleasures, seamen’s bodies were regulated through measured food and drink and morality kept under surveillance by restricted mobility and insistence on sport and reading. The Calcutta Sailors’ Home authorities requested the police commissioner not to issue licence for grog shops near the Sailors’ Home without government approval.^94^ V.H. Schalch, Chairman of the Justices, told Major Malleson that the Sailors’ Home and boarding houses in Calcutta were comfortable, but the latter often caused problems by asking seamen to deposit a week’s rent and pay for a bottle of liquor at the time of checking in, failing to do which seamen had to pawn their clothes. Since seamen were paid in an advance note that could not be readily cashed, they had to dig into their savings that often ran out quickly. Merely a ‘night’s debauch’ emptied their pockets, and they landed in hospital or prison unless they managed to secure a job promptly afterwards.^95^ Malleson commented that many boarding masters encouraged seamen to keep drinking on their premises, and drained them out of their savings.^96^

The Sanitary Commission rejected Malleson’s recommendation of enforcing the British Vagrancy Act in Calcutta to arrest unruly seamen, and abolishing privately-owned boarding houses in favour of state-controlled accommodation for seamen.^97^ The Commission rather insisted on appointing a Marine or Port Magistrate, better regulation for shore leave for seamen, and establishment of riverside dispensaries. The Bengal Government was supportive of the Sailors’ Home, even saying that the institution was unique in treating seamen like ‘rational’ beings.^98^ The annual report of the Sailors’ Home in 1873 revealed the continuance of the crimping problems, using more or less the same vocabulary as 40 years before. While more seamen had opted for meals at the Sailors’ Home, crimps were still on the lookout to ‘inveigle’ seamen to the Flag Street neighbourhood. In 1869 the number of eaters was 175; in 1870, 603; in 1871, 636; in 1872, 575; and in 1873, 732.^99^

The Methodist Church opened a Seamen’s Reading and Coffee Rooms in Lal Bazar in 1874. Apart from preaching abstinence during their nightly religious service for visiting seamen, they encouraged teetotalism by offering coffee and refreshment at low prices. The Coffee Rooms also had a reading hall with newspapers, magazines and about 500 books gifted by ‘friends of the institution.’ Some European women invited seamen to tea at their homes, a practice that began in the late 1850s to thank seamen for helping resident Europeans during the Sepoy Mutiny.^100^ In 1892, the government made an annual contribution of 3,000 rupees and the city’s merchants donated 3,500 rupees to help run the institution. The main agents of the institution were a group of women including Mrs Meik, Mrs Conklin and Mrs Henderson, who visited grog shops in the Sunday afternoons and invited seamen to the services.^101^ Their involvement shows that Methodist women missionaries were active not only in local women’s education and health, but also in the welfare of poor Europeans. Rev. Frank W. Warne quoted George Henderson, the Coffee Rooms manager, saying in 1891 that seamen greatly appreciated the refreshment rooms at the mission.^102^ He wrote in 1898 that the self-supporting Sailors’ Home provided about 10,000 free meals each year to destitute seamen.^103^ It is not known if seamen followed the dietary evangelicalism voluntarily, or were just paying lip service to the missionaries’ self-evaluation as excellent reformers for the cheap or free meals. Either way, they were subject to a regulated diet designed to improve their morality. The lead in seamen’s welfare seemed to have shifted to the Methodist mission from the Baptist Church in the last quarter of the nineteenth century.

In 1883, *The Times of India* reported an ‘epidemic’ of mutiny and desertion among seamen in Calcutta. In a meeting at the Spencer’s Hotel on 13 July, shipowners identified as the intention to escape the law in England and the judicial leniency with cases of desertion. Capt. Smith of the British ship *Dawpool* criticised the popular perception of ‘poor Jack’ as the victim of circumstances, saying ship owners were no less affected by seamen’s behaviour. At another meeting, held at the Dalhousie Institute on 17 July, ship captains discussed how effectively seamen could be dissuaded from malingering and deserting.^104^ These discussions did not result in much improvement of seamen’s employment situation. British seamen in Calcutta and other Indian ports never ceased to complain of severe hardship at work, especially being forced to do what they were not suitable for, thus endangering themselves and the lives of their co-workers. The Seamen’s Friendly Society delivered a memorial to the President of the Board of Trade, urging him to enquire into maritime labour problems.^105^ One observer wrote that Christian philanthropic organisations could not fully reduce seamen’s distress. In the 1880s, the Sailors’ Home officials allegedly colluded with crimps. He reported that the Benedictine clergy Father Hopkins of the Priory and Capt. J.H. Williams managed to overhaul the management after a long struggle.^106^ Apparently, Benedictine clergy followed Baptist and Methodist missionaries in seamen’s welfare, though they never collaborated. The statistics of the Presidency General Hospital in the 1890s encapsulated the outcome of the struggle, revealing the seamen’s quarters to have remained Calcutta’s syphilis hotspot.^107^ In some ways, missionaries were more dynamic than the EIC government and the British colonial state in curbing drinking and desertions problems by getting sober seamen employment on ships. Their strange alliance with the state was articulated probably the most aphoristically by Norman Chevers. If a healthy sailor asked him the secret of longevity, he would tell him to be moral and religious.^108^ Local governments helped missionaries by allowing them to use abandoned vessels and building for their work, but they rarely collaborated in regulating or reforming seamen.

## Conclusion

This article has examined the efforts of British Baptist missionaries to regulate white European seamen’s health and habits in nineteenth-century India as part of their global effort of making ideal Christians and imperial subjects. Calcutta’s importance in the network of ecclesiastical missions was evident from the establishment of a Sailors’ Home in the city after London and New York. The Seamen’s Mission in India grew out of the Christian missionaries’ anxiety and moral obligation to protect British people’s health and by extension racial superiority in a debasing climate. Their actions were similar to the measures to dissuade soldiers from drinking and consorting with prostitutes in cantonments. A key difference was the missionaries’ hegemonic emphasis on self-discipline and the veiling of moral regulation in a garb of benevolence and goodwill. This image of religious altruism dismantles on a close examination, as the Sailors’ Home was a site of controversy and missionaries sometimes accused each other of malpractice. The article has pointed out that the effectiveness of Sailors’ Homes was more asserted than demonstrated. Rather than evaluate the efforts of missionaries, it has analysed the entanglement of religion, racial identity, and imperial politics in philanthropic institutions.

The local government often helped seamen’s missions by offering them buildings or plots of land. Sailors’ Homes in the nineteenth century were remarkable as meeting points of religious, political and commercial actors on a humanitarian ground. The nature of imperial collaboration expressed through the services of these institutions indicates the existence of an organised network of governance. This is not to suggest that colonial governance was highly centralised or colonial society followed a chain of command, as we have seen missionaries and colonial officials often failing to understand and arguing about the most desirable and effective ways of taking care of seamen. The state was not very effective and sometimes lacked any real motivation to protect seamen, notwithstanding the massive commercial implication of the latter’s good health. Their ventures were taken forward mostly by employees with private interests rather than any systematic investment in resources. The network, however imperfect, shows that the British in positions of power and wealth in India were willing to act as benefactors of their less fortunate countrymen.

## Notes

1. ‘Local Intelligence: Bombay’, *Parbury’s Oriental Herald* 1, no. 1 (1838), p. 546.

2. G.B. Malleson, ‘The State of the Sailors in Calcutta’, British Library IOR: P/437/29, Government of India, Home Department Proceedings, Marine 1866, No. 18, p. 20.

3. Ashwini Tambe, *Codes of Misconduct: Regulating Prostitution in Late Colonial Bombay* (Minneapolis and London: University of Minnesota Press, 2009), p. 27.

4. The sailortown, a term coined by sailor and folk musician Stan Hugill, was representative of a liminal urban space for both mobile and sedentary seamen, and notorious for their squalor and disorderly nature. The distinctive features of these spaces were health and sanitation problems, dynamic relationships between various racial and occupational groups, and the urban and maritime elite’s constant effort to segregate them from the more respectable areas of the city. Stan Hugill, *Sailortown* (London: Routledge and Kegan Paul, 1967), 312. For explorations into sailortown culture see Valerie Burton, ‘Boundaries and Identities in the Nineteenth-Century English Port: Sailortown Narratives and Urban Space’, in Simon Gunn and Robert J. Morris (eds), *Identities in Space: Contested Terrains in the Western City since 1850* (Aldershot: Ashgate, 2001), pp. 137–51; Graeme J. Milne, *People, Place and Power on the Nineteenth-Century Waterfront: Sailortown* (Basingstoke: Palgrave Macmillan, 2016); Brad Beaven and Mathias Seiter, ‘Regulating Sin in the City: The Moral Geographies of Naval Port Towns in Britain and Germany, c. 1860–1914ʹ, *Britain and the World* 13, no. 1 (2020), pp. 27–46.

5.
Some of the important works are Gopalan Balachandran, ‘Recruitment and Control of Indian Seamen: Calcutta, 1880–1935', *International Journal of Maritime History* 9, no. 1 (1997), pp. 1–18; Ravi Ahuja, ‘Mobility and Containment: The Voyages of South Asian Seamen, c. 1900–1960ʹ, *International Review of Social History* 51, no. S14 (2006), pp. 111–141; Michael H. Fisher, ‘Finding Lascar “Wilful Incendiarism”: British Ship-Burning Panic and Indian Maritime Labour in the Indian Ocean’, *South Asia: Journal of South Asian Studies* 35, no. 3 (2012), pp. 596–623; Aaron Jaffer, *Lascars and Indian Ocean Seafaring, 1780–1860: Shipboard Life, Unrest and Mutiny* (Woodbridge: Boydell & Brewer, 2015).

6. Harald Fisher-Tiné, ‘Flotsam and Jetsam of the Empire?: European Seamen and Spaces of Disease and Disorder in Mid-nineteenth Century Calcutta’, in Ashwini Tambe and Harald Fisher-Tiné (eds), *The Limits of British Colonial Control in South Asia: Spaces of Disorder in the Indian Ocean Region* (London and New York: Routledge, 2009), pp. 121–54; Sarmistha De, *Marginal European in Colonial India, 1860–1920* (Kolkata: Thema, 2008). The idea of destitute seamen causing the ruling class enough embarrassment for the latter to mobilise its legal resources and social welfare schemes to the benefit of seamen is also articulated by Constance Mary Turnbull with reference to Singapore. C.M. Turnbull, *A History of Modern Singapore, 1819–2005* (Singapore: NUS Press, 2009), pp. 80–81.

7. David Arnold, ‘European Orphans and Vagrants in India in the Nineteenth Century’, *The Journal of Imperial and Commonwealth History* 7, no. 2 (1979), pp. 104–27; Elizabeth Buettner, ‘Problematic Spaces, Problematic Races: Defining “Europeans” in Late Colonial India’, *Women’s History Review* 9, no. 2 (2000), pp. 277–98; Harald Fischer-Tiné, *Low and Licentious Europeans: Race, Class and ‘White Subalternity’ in Colonial India* (New Delhi: Orient Blackswan, 2009); Erica Wald, *Vice in the Barracks: Medicine, the Military and the Making of Colonial India, 1780–1868* (Basingstoke; Palgrave Macmillan, 2014).

8. Roald Kverndal, *Seamen’s Missions: Their Origin and Early Growth* (Pasadena, CA: William Carey Library, 1986); Roald Kverndal, *The Way of the Sea: The Changing Shape of Mission in the Seafaring World* (Pasadena, CA: William Carey Library, 2008); Roald Kverndal, *George Charles Smith of Penzance: From Nelson Sailor to Mission Pioneer* (Pasadena, CA: William Carey Library, 2012); Alston Kennerley, *British Seamen’s Missions and Sailors’ Homes 1815–1970: Voluntary Welfare Provision for Serving Seafarers*, unpublished PhD thesis, University of Plymouth, 1989; Alston Kennerley, ‘Seafaring Missionary Societies and Maritime Education and Training, 1815–1914ʹ, *Paedagogica Historica* 35, sup 1 (2015), pp. 241–57; Robert Miller, *One Firm Anchor: The Church and the Merchant Seafarer* (Cambridge: Lutterworth Press, 2012); Richard Blake, *Evangelicals in the Royal Navy 1775–1815: Blue Lights & Psalm-Singers* (Woodbridge: Boydell Press, 2008); Richard Blake, *Religion in the British Navy 1815–1879: Piety and Professionalism* (Woolbridge: Boydell Press, 2014); Stephen Friend, *Fishing for Souls: The Development and Impact of British Fishermen’s Missions* (Cambridge: Lutterworth Press, 2018).

9. See Jeffrey Cox, *Imperial Fault Lines: Christianity and Colonial Power in India, 1818–1940* (Stanford, CA: Stanford University Press, 2002); Robert Eric Frykenberg (ed), *Christians and Missionaries in India: Cross-Cultural Communication since 1500* (London and New York: Routledge, 2003); Hayden J.A. Bellenoit, *Missionary Education and Empire in Late Colonial India, 1860–1920* (London: Pickering & Chatto, 2007); Parna Sengupta, *Pedagogy for Religion: Missionary Education and the Fashioning of Hindus and Muslims in Bengal* (Berkeley: University of California Press, 2011); David Hardiman, *Missionaries and Their Medicine: A Christian Modernity for Tribal India* (Manchester: Manchester University Press, 2017); and Sutapa Dutta, *British Women Missionaries in Bengal, 1793–1861* (London: Anthem Press, 2017).

10. Satoshi Mizutani, *The Meaning of White: Race, Class, and the ‘Domiciled Community’ in British India 1858–1930* (Oxford: Oxford University Press, 2011); Onni Gust, *Unhomely Empire: Whiteness and Belonging, c. 1760–1830* (London: Bloomsbury, 2021).

11. Darcy Ingram, ‘Saving the Union’s Jack: The Montreal Sailors’ Institute and the Sailors’ Homeless Sailor, 1862–98ʹ, in Bettina Bradbury and Tamara Myers (eds), *Negotiating Identities in 19th- and 20th-Century Montreal* (Vancouver and Toronto: UBC Press, 2005), pp. 49–76.

12. David Roberts, *Paternalism in Early Victorian England* (London: Croom Helm, 1979).

13. ‘The Eighth Anniversary Meeting’, *The Sailors’ Magazine* 3, no. 30 (June 1841), p. 182.

14. Charles Rosenberg, ‘Florence Nightingale on Contagion: The Hospital as Moral Universe’, in *Explaining Epidemics and Other Studies in the History of Medicine* (New York: Cambridge University Press, 1992), p. 94.

15. ‘Anniversary of the Calcutta Seamen’s Friend Society, Calcutta, Dec. 21, 1839ʹ, *The Sailors’ Magazine* 12, no. 12 (August 1840), p. 369.

16. Adele Perry, *On the Edge of Empire: Gender, Race, and the Making of British Columbia, 1849–1871* (Toronto: University of Toronto Press, 2001), p. 84.

17. ‘Heart Cheering News from Calcutta, East Indies’, *The Mariners’ Church Gospel Temperance Soldiers’ and Sailors’ Magazine* 25, no. 10 (October 1845), p. 440.

18. Kverndal, *Seamen’s Missions*, p. 246. For an account of Carey’s important work as missionary, translator and social reformer, see Daniels Potts, *British Baptist Missionaries in India 1793–1837: The History of Serampore and Its Missions* (Cambridge: Cambridge University Press, 1967).

19. ‘Calcutta Bethel Society’, *The Friend of India* 7 (Serampore: Mission Press, 1824), p. 349.

20. Kverndal, *Seamen’s Missions*, p. 291.

21. Esme Cleall, *Missionary Discourses of Difference: Negotiating Otherness in the British Empire, 1840–1900* (Basingstoke: Palgrave Macmillan, 2012), p. 145.

22. ‘The Sailors’ Home’, *The Calcutta Christian Observer* 6, no. 58 (1837), p. 132.

23. Ibid, p. 133.

24. Ibid.

25. The naval authorities undertook regulation of diet, provision of clean habitat, and eradication of sources of miasma and insanitary living conditions in the second half of the nineteenth century. Marine Department’s intervention in medical matters was arguably inadequate for three reasons: its lack of scientific expertise, the reluctance of ship owners to curtail their profit and provide better welfare to seamen, and seamen’s failure to articulate their needs. Tim Carter, *Merchant Seamen’s Health*, pp. 70–84.

26. In Conrad Dixon and Sarah Palmer’s analysis of the relationship of seamen with boarding housekeepers and crimps, the latter has been positioned as an element in the labour market rather than the malicious beings as contemporary records portrayed them. Dixon, ‘The Rise and Fall of the Crimp, 1840–1914ʹ, in Stephen Fisher (ed), *British Shipping and Seamen, 1630–1960: Some Studies* (Exeter: University of Exeter Press, 1984), pp. 49–67; Palmer, “Seamen Ashore in Late Nineteenth Century London: Protection from the Crimps”, in *Seamen in Society*, ed. Paul Adam (Bucharest: International Commission on Maritime History, 1980), 55–67. Alston Kennerley’s writings about Sailors’ Homes and Seamen’s Missions movements have examined how the British society, especially the Christian Church, sought to protect seamen from crimps. Kennerley, ‘British Seamen’s Missions in the Nineteenth Century’, in Lewis R. Fischer et.al. (ed), *The North Sea: Twelve Essays on Social History of Maritime Labour* (Stavanger: Stavanger Maritime Museum, 1992), pp. 79–98.

27. Major Malleson singled this category of crimps as a phenomenon found only in Calcutta. These boatmen were allowed to board ships and sell liquor and ‘unwholesome’ food items. The Preventive Officer was instructed to overlook these bumboats. Malleson, ‘The State of the Sailors in Calcutta’, p. 11.

28. John Statham, *India Recollections* (London: Samuel Bagster, 1832), p. 10.

29. Ibid, p. 10.

30. ‘Miscellanious Notices’, *Calcutta Review* 9, no. 18 (January-June 1848), p. xlv.

31. ‘The Crimping System- No.1ʹ, *The Sailors’ Magazine* 17, no. 2 (October 1844), pp. 116–19.

32. Michael Macilwee, *The Liverpool Underworld: Crime in the City, 1750–1900* (Liverpool: Liverpool University Press, 2011).

33. *The Sailors’ Magazine* (January 1845), pp. 139–41.

34. *The Friend of India*, 30 August 1866.

35. Ibid.

36. ‘The Crimping System in London’, *The Sailors’ Magazine* 17, no. 5 (January 1845), pp. 139–41.

37. ‘Sailors’ Home Society’, *The Calcutta Monthly Journal* 4 (February 1838), p. 98.

38. *The Bengal Hurkaru* (30 January 1824), p. 203.

39. British Library IOR, Government of India, Fort William Marine Paymaster’s Office. Home: Public; P.P. 2789–2794, O.C., 24 April., No. 8; 23 April 1789. Letter from Mr. J. Price, Marine Paymaster, to Mr. E. Hay, Secretary, suggesting a plan for maintaining and utilising the services of vagrant seamen.

40. *Selections from Calcutta Gazettes*, I vol, W.S. Seton-Karr (ed.) (Calcutta: O.T. Cutter and Military Orphan Press, 1864), p. 242.

41. *Calcutta Gazette*, 30 September 1790, *Selections from Calcutta Gazettes*, II vols, W.S. Seton-Karr (ed.) (Calcutta: O.T. Cutter and Military Orphan Press, 1865), p. 274.

42. *Calcutta Gazette*, 20 November 1794, *Selections from Calcutta Gazettes*, II vols, W.S. Seton-Karr (ed.) (Calcutta: O.T. Cutter and Military Orphan Press, 1865), p. 397.

43. *Bengal Hurkaru*, 1 December 1857, p. 238.

44. Major H. Hobbs, *John Barleycorn Bahadur: Old Time Taverns in India* (Calcutta: Thacker, Spinck & Co, 1943), p. 100.

45. Sumanta Banerjee, ‘‘City of Dreadful Night’: Crime and Punishment in Colonial Calcutta,’ *Economic and Political Weekly* 38, no. 21 (2003), p. 2048.

46. Hobbs, *John Barleycorn Bahadur*, p. 134.

47. ‘Calcutta Sailors’ Home’, *The Calcutta Christian Observer* 6, no. 59 (April 1837), p. 208.

48. For a study of how missionaries racialised the problem of low quality provisions and the liquor sold in Indian markets, see Manikarnika Dutta, ‘European Sailors, Alcohol, and Cholera in Nineteenth-Century India’, in Gwyn Campbell and Eva-Maria Knoll (eds), *Disease Dispersion and Impact in the Indian Ocean World* (New York: Palgrave Macmillan, 2020), pp. 190–210.

49. ‘Sailors’ Home’, *The Calcutta Christian Observer* 6, no. 61 (June 1837), p. 319.

50. ‘Calcutta Sailors’ Home’, *Madras Missionary Register* 4, no. 1 (January 1838), pp. 122–23.

51. Barrett, *Calcutta: Strange Memoirs*, pp. 391–93.

52. *The Sailor’s Magazine* 10, no. 8 (1838), p. 362.

53. It also inspired a group of preachers to convene a meeting at the ‘American hong’ on 3 January 1838 that led to the establishment of the Seamen’s Friend Association in China. ‘Seamen’s Friend Association’, *The Chinese Repository* 7, no. 9 (January 1839), pp. 477–78.

54. *The Bengal Hurkaru*, 10 December 1842, p. 1.

55. *The Calcutta Christian Observer* (September 1841), pp. 590–91.

56. For a useful study of the formulaic nature of letters from subordinates to superiors, see Martyn Lyons, ‘Writing Upwards: How the Weak Wrote to the Powerful’, *Journal of Social History* 49 no. 2 (2015), pp. 317–30.

57. ‘Calcutta Sailors’ Home’, *Madras Missionary Register* 4, no. 1 (January 1838), p. 123.

58. *16th Annual Report of the Calcutta Sailors’ Home* (Calcutta: Military Orphan Press, 1854), pp. 5–6.

59. Ibid, pp. 8–9.

60. Chieko Ichikawa, ‘Writing as Female National and Imperial Responsibility: Florence Nightingale’s Scheme for Social and Cultural Reforms in England and India’, *Victorian Literature and Culture* 39 (2011), p. 93.

61. Florence Nightingale, letters to Douglas Galton dated 29 January 1864 and 9 February 1864, in Gérard Vallée (ed), *Florence Nightingale on Health in India* (Waterloo, Ontario: Wilfrid Laurier University Press, 2006), pp. 485–86.

62. Florence Nightingale, letter to Sir John Lawrence dated 26 September 1864, in Ibid, p. 498.

63. Charles Rathbone Low, *History of the Indian Navy: (1613–1863)* (London: Richard Bentley and Son, 1877), p. 572.

64. *Dundee, Perth, and Cupar Advertiser*, 5 September 1851, p. 3.

65.
From S.H. Robinson, Secretary Sailors’ Home to Lieutenant colonel H.C James, Private Secretary to the Lieutenant-Governor of Bengal, (dated 25 February 1864), 5, Marine Department, West Bengal State Archives.

66.
From F.R. Cockerell, Officiating Secretary to the Government of Bengal, to S.H Robinson, Secretary, Sailors’ Home, (no. 1146, dated 5 March 1864), 5, Marine Department, WBSA.

67. *Annual Report on the Administration of the Bengal Presidency for 1863–64* (Calcutta: Military Orphan Press, 1865), p. 58.

68. ‘Opening of the New Sailors’ Home in Calcutta’, *Homeward Mail from India, China and the East*, 20 February 1868, p. 5.

69. ‘Heart Cheering News from Calcutta, East Indies’, *The Mariners’ Church Gospel Temperance Soldiers’ and Sailors’ Magazine* 25, no. 10 (October 1845), p. 451.

70. *The Sailors’ Magazine* 16, no. 4 (1843), p. 105.

71. ‘Heart Cheering News from Calcutta, East Indies’, *The Mariners’ Church Gospel Temperance Soldiers’ and Sailors’ Magazine* 25, no. 10 (October 1845), p. 448.

72. ‘British India Pleading for Sailors’ Missionary Exertions’, *The Mariners’ Church Gospel Temperance Soldiers’ and Sailors’ Magazine* 25, no. 10 (October 1845), p. 442.

73. ‘Calcutta Seamen’s Friend Society’, *The Sailors’ Magazine* 24, no. 2 (October 1851), p. 426.

74. *The Sailors’ Magazine* 29, no. 8 (April 1857), p. 251.

75. ‘A Voice from the Sea’, *The Sailors’ Magazine and Seamen’s Friend* 51, no. 1 (January 1879), p. 15.

76. Susan Thorne, *Congressional Missions and the Making of an Imperial Culture in Nineteenth-Century Britain* (Stanford: Stanford University Press, 1999), pp. 35–36.

77. *16th Annual Report of the Calcutta Sailors’ Home* (Calcutta: Military Orphan Press, 1854), pp. 5–6.

78. Barrett, *Calcutta: Strange Memoirs – Foreign Perceptions*, pp. 391–93.

79. Thomas Atkins, *Reminiscences of Twelve Years in Tasmania and New South Wales, Norfolk Island and Moreton Bay; Calcutta, Madras and Cape Town; The United States if America and the Canadas* (Malvern: Advertiser Office, 1869), p. 91.

80. Ibid, 92. The daily price of accommodation was nine-pence for sailors, a shilling for petty officers, and eighteen-pence for chief officers. The residents were asked to deposit their entire pool of money to Atkins and allowed to draw one rupee per day to meet their expenses. Many seamen flouted this regulation, some of them preferring punch shops to the Sailors’ Home, repudiating the very existence of the shelter.

81. Ibid, p. 94.

82. Ibid, p. 95.

83. ‘Miscellanious Notices’, *Calcutta Review* 9, no. 18 (January-June 1848), p. xlvi.

84. Ibid., p. xlvi.

85. ‘Seamen in Calcutta’, *The Shipping and Mercantile Gazette*, 15 November 1853, p. 8.

86. Montague Massey, *Recollections of Calcutta for over Half a Century* (Calcutta: Thacker, Spinck, 1918), p. 89.

87. Chevers, *On the Preservation of the Health of Seamen in Calcutta*, p. 46.

88. *The Statesman*, 5 December 1882, p. 1705.

89. Malleson, ‘The State of the Sailors in Calcutta’, pp. x–xiii.

90. David Beckingham, *The Licenced City: Regulating Drink in Liverpool, 1830–1920* (Liverpool: Liverpool University Press, 2017), p. 1.

91. Ibid, pp. 103–4.

92. Ibid, p. 115.

93. ‘A Visit to the Sailors’ Home at Portsmouth’, *The Leisure Hour*, no. 129 (15 June 1854), pp. 382–83.

94. Revenue Proceedings No. 21, May 1867, Miscellaneous Revenue Department, Government of Bengal, WBSA.

95. Malleson, ‘The State of Sailors in Calcutta’, p. 10.

96. Ibid, p. 11.

97. British Library IOR: P/437/29, Government of India, Home Department Proceedings, Marine 1866, No. 18, Letter No. 115, J.M. Cunningham, Officiating Secretary to the Sanitary Commission for Bengal to A. Eden, Secretary to the Government of Bengal, 9 February 1866, p. 118.

98. *Annual Report of the Sanitary Commissioner for Bengal for 1868* (Calcutta: Alipore Jail Press, 1869), p. 561.

99. The daily rate of room and board (breakfast and three substantial meals) was 12 annas (1s. 6d.), which compared favourably with the daily rates in Sydney (2s. 6d.) and Melbourne (3s.), and the weekly rates in Liverpool (16s.) and London (15s.). ‘Calcutta Sailors’ Home’, *Sunderland Daily Echo and Shipping Gazette*, 23 September 1874, p. 4.

100. Hugill, *Sailortown*, p. 316.

101. *74th Annual report of the Missionary Society of the Methodist Episcopal Church for the Year 1892* (New York, 1893), p. 222.

102. *73rd Annual report of the Missionary Society of the Methodist Episcopal Church for the Year 1891* (New York, 1892), p. 202.

103. *90th Annual report of the Missionary Society of the Methodist Episcopal Church for the Year 1898* (New York, 1899), p. 229.

104. *The Times of India*, 19 July 1883, p. 3.

105. ‘Grievances of British Seamen’, *The Globe*, 2 June 1894, p. 3.

106.
Hugill, *Sailortown*, p. 315.

107. J.G. Pilcher, *Report on the Calcutta Medical Institutions for the Year 1892* (Calcutta: Bengal Secretariat Press, 1893), p. 9.

108. Chevers, *On the Preservation of the Health of Seamen*, p. 48.

